# Physicochemical and Functional Properties of Membrane-Fractionated Heat-Induced Pea Protein Aggregates

**DOI:** 10.3389/fnut.2022.852225

**Published:** 2022-03-23

**Authors:** Nancy D. Asen, Rotimi E. Aluko

**Affiliations:** ^1^Department of Food and Human Nutritional Sciences, University of Manitoba, Winnipeg, MB, Canada; ^2^The Richardson Center for Functional Foods and Nutraceuticals, University of Manitoba, Winnipeg, MB, Canada

**Keywords:** pea protein, heat treatment, protein aggregates, polypeptide composition, pH, surface hydrophobicity, functional properties

## Abstract

This study was carried out to investigate the effect of heat pre-treatment of pea proteins at different pH values on the formation of functional protein aggregates. A 10% (w/v) aqueous mixture of pea protein concentrate (PPC) was adjusted to pH 3.0, 5.0, 7.0, or 9.0 followed by heating at 100°C for 30 min, cooled and centrifuged. The supernatant was sequentially passed through 30 and 50 kDa molecular weight cut-off membranes to collect the <30, 30–50, and >50 kDa fractions. The >50 kDa fractions from pH 3.0 (FT3), 5.0 (FT5), 7.0 (FT7), and 9.0 (FT9) treatments had >60% protein content in contrast to the ≤20% for the <30 and 30–50 kDa fractions. Therefore, the >50 kDa fractions were collected and then compared to the untreated PPC for some physicochemical and functional properties. Protein aggregation was confirmed as the denaturation temperature for FT3 (124.30°C), FT5 (190.66^o^C), FT7 (206.33^o^C) and FT9 (203.17^o^C) was significantly (*p* < 0.05) greater than that of PPC (74.45^o^C). Scanning electron microscopy showed that FT5 had a compact structure like PPC while FT3, FT7, and FT9 contained a more continuous network. In comparison to PPC, the >50 kDa fractions showed improved solubility (>60%), oil holding capacity (~100%), protein content (~7%), foam capacity (>10%), foam stability (>7%), water holding capacity (>16%) and surface hydrophobicity (~50%). Least gelation concentration of PPC (18%), FT3 (25%), FT5 (22%), FT7 (22%), and FT9 (25%) was improved to 16, 18, 20, 16, and 18%, respectively, after addition of NaCl.

## Introduction

Utilization of protein as a food ingredient and additive is an age long global tradition and this is seen in wide applications from small scale (e.g., private home kitchens) to industrial scale food processing. Protein is valued by food formulators and the industry for properties such as gelation (e.g., pasta and sausages), foam (e.g., meringues and cakes), emulsification (e.g., salad dressings and soups), fat holding (e.g., yogurts and fish meat products), and water binding (e.g., meat and bread) (1). In addition, proteins contribute nutritional benefit to foods through amino acids, which are essential for human growth and preservation. Food proteins are derived from plant and animal sources with the most popular being meat, poultry, seafood, beans, peas, lentils, eggs, nuts, seeds, and soy. The recent trend in food applications is the exploration of plant proteins as alternatives to animal proteins, especially to meet the triad of health, socioeconomic and environmental demands in addition to the challenge to produce and distribute quality protein to feed >9 billion people by 2050 (2). The reason for this trend is that plant proteins have several advantages over animal equivalents, including higher levels of unsaturated fatty acids, low cost, ready availability, low greenhouse gas emission, reduced carbon footprint and an alternative protein source for vegetarians and vegans (3, 4). As a result, high consumer preference for plant proteins as alternative protein source has been discussed (5). One of the most commercially utilized plant proteins comes from the yellow field pea seed. In addition to the benefits of plant proteins, and unlike other grain proteins from wheat and soybean, pea proteins are less allergenic and possess nitrogen fixing ability, which makes it an important agricultural crop for rotation to maintain soil health (6, 7). Several works have shown the potential use of pea protein as a food ingredient to improve emulsification, gelation, and foaming properties with possible development of novel foods like beverages, baked goods, soups, snacks, dips, and salad dressings (8, 9). Peas have been a popular source of protein in developing countries for decades, however, until recently, consumption as part of manufactured food products have been very limited mainly due to availability of many affordable alternative protein sources like soybean and milk.

The main pea proteins are the globulins (55–65%) but albumins, prolamin, and glutenins are also present. The globulin fraction consists mainly of legumin (11S), vicilin (7S), and convicilin (7S). Legumins have three acidic subunits and three basic subunits with molecular weight (MW) of 36.8, 36.4, and 34.4 kDa for acidic and ~20 kDa for basic subunits. The vicilin β + γ subunit has MW of 23.6–31.6 kDa while vicilin basic subunit (α + β + γ) has MW 54.6 kDa and subunit γ is 17.4 kDa in size (10). Two-dimensional gel electrophoresis of pea seed albumins showed three groups, high molecular weight (~50–110 kDa), average molecular weight (~20–35 kDa) and low MW (~ 6–17 kDa) (10, 11). About 638 amino acids were found in the protein primary structure of different pea seed cultivars (12). Pea proteins contain a wide variety and well-balanced profile of amino acids belonging to different classes of positively charged, negatively charged, branched-chain, aromatic and hydrophobic (9). However, previous works have shown that like other legumes, sulfur-containing amino acids (methionine and cysteine) are limiting in pea proteins (13, 14). The main amino acid in pea legumin α-chain is Glu, while the β-chain is rich in Val, Ala, and Leu and presence of high levels of Glu in proteins enhance protein-solvent interactions, which improves functionality (15). However, the globular nature of pea proteins hinders their flexibility as the structure is densely packed due to the presence of disulfide linkages, low surface charge density, hydrophobic effects, hydrogen bonds, electrostatic and van der Waals forces, which ultimately impairs solubility and other protein functionalities (16–19). Furthemore, the secondary structure of pea proteins is higher in the rigid β-sheet (30–41%), which contributes to the low solubility when compared to animal proteins (egg white and whey) with lots of the flexible α-helical structure (17–19). Inherent properties like beany flavor and the presence of antinutritional materials in pea protein also reduce its quality and value as a food ingredient (20). As a result of this complex conformation, globular proteins require some degree of pre-treatment to isolate specific fractions with enhanced functionality.

Techniques engaged in the modulation of protein structure could be largely classified as physical (ultrasound, thermal, high pressure), chemical (glycosylation, complex formation), and enzymatic. Heat treatment is a relatively cheap and simple method employed for food processing and preservation in which all the hierarchical structures of the protein could be affected (21). Literature is replete with studies where soybean and milk protein are thermally treated using a variety of methods. The effect of ohmic heat (17, 23, 30, and 37 V/cm) on structural and functional properties of soybean protein was studied and the outcome was improved amino acid content and emulsification activity index by 14 and 38%, respectively, while foaming properties, emulsion stability index, sulfhydryl content, and surface hydrophobicity declined (22); however, the study reported no significant structural changes in the protein. Pre-heating at 90°C for 2.5 min improved solubility, surface hydrophobicity, and the gelling properties of soy protein isolate and soy-egg composite gels (23). The significant increase in solubility was attributed to the formation of soluble macro-complexes. A functional whey protein powder with high water binding capacity was produced by addition of lactose at pH 10 and dry heating at 120°C for >30 min (24). Ryan and Foegeding (25) reported the presence of soluble whey protein aggregates after pre-treatment at 90°C for 10 min at pH 7.5. A few works have also been reported in literature about thermal treatment of pea protein. The application of heat to pea proteins at slightly above the denaturation temperature (>82°C) has been suggested to improve flexibility of the molecules (26). Another study on the solubility and heat stability of pea protein isolate showed high solubility (>92%) after heat treatment at 121°C for 2.8 min because of the formation of soluble aggregates and increased heat stability (15). During denaturation, pea protein subunits dissociate, and the structure unfolds to form aggregates at elevated temperatures. Some of the aggregates formed at elevated temperatures are stabilized by disulfide linkages and hydrophobic interactions (27, 28). Previous works have focused on the effect of heat pretreatment on functional and physicochemical properties of the native protein concentrate or isolate with or without pH adjustments and without fractionation. However, there is the need to evaluate the effect of pH and protein size on the properties of heat-induced protein aggregates. Therefore, the aim of this work was to determine the structural and functional properties of >50 kDa pea protein aggregates prepared at different pH values and isolated by membrane ultrafiltration. The aggregates were compared with the native unaggregated pea protein concentrate to measure potential relevance as ingredients for the food industry.

## Materials and Methods

### Materials and Chemicals

The yellow field pea protein concentrate (PPC) was purchased from Nutri-Pea Limited (Portage La Prairie, MB, Canada). Other chemical reagents used were purchased from Sigma-Aldrich (St. Louis, MO, USA) and Fisher Scientific Company (Oakville, ON, Canada). All the chemicals and reagents were of high purity analytical grade with double distilled water used for their preparation.

### Preparation of Protein Aggregates

A 10% (w/v) mixture of the PPC (68.6% protein content) was prepared using distilled water and stirred for 10 min. The mixture was then adjusted to pH 3.0, 5.0, 7.0, or 9.0 using 0.1 M NaOH or 0.1 M HCl and the containers hermetically sealed to allow for maximum heat penetration in the mixture followed by immersion in a shaking water bath at 100°C for 30 min. After cooling rapidly in an ice bath to ~20°C, the heated protein mixture was centrifuged at 7,000 × *g* for 30 min and the supernatant saved while precipitate was discarded.

### Fractionation of the Protein Aggregates

The supernatants obtained from the heated protein mixtures were each sequentially passed through Amicon®stirred ultrafiltration cell fitted with a 30 kDa molecular weight cut-off (MWCO) membrane and the permeate collected as the < 30 kDa fraction. The retentate was mixed with an equal amount of distilled water and passed through a 50 kDa MWCO membrane (coupled with diafiltration) and the permeate collected as 30–50 kDa fraction while the retentate was labeled as >50 kDa fraction. The three fractions were freeze-dried and stored at −20^o^C. The PPC and freeze-dried ultrafiltration fractions were dissolved in 0.1 M NaOH, and total protein content determined using the Lowry method (29). Based on the low protein contents of the < 30 and 30–50 kDa fractions, only the freeze dried >50 kDa fractions, which had a high protein content like the PPC were used to perform all the following experiments. All the analysis were carried out in triplicates and reported as mean and standard deviation.

### Protein Solubility, Content, and Yield

Protein solubility of the >50 kDa aggregated proteins was determined as previously described (30). Briefly, 10 mg of the protein was suspended in 1 mL buffers (pH 3.0–9.0) followed by vortexing and centrifugation at 10,000 × *g* for 20 min. The protein content of the supernatant was determined with bovine serum albumin as the standard (29) and expressed as percentage ratio of the total protein to obtain protein solubility values. The gross protein yield was analyzed as described by Famuwagun et al. (31).

### Surface Hydrophobicity

The surface hydrophobicity of the samples was determined according to the method described by Haskrad and Li-Chan (32). The stock sample was prepared using 10 mg/mL in 10 mM phosphate buffer, pH 7.0. The mixture was vortexed, kept for 1 h and then centrifuged at 10,000 × *g* for 10 min at 25^o^C. Serial dilutions of the supernatant were prepared to obtain 0.5–2.5 mg/mL protein concentrations. Using a 96 well-microplate, 5 μL of 8 mM l-anilinonaphthalene-8-sulphonate (ANS) in 10 mM phosphate buffer (pH 7.0) was added to 200 μL of sample followed by fluorescence intensity (FI) measurement at excitation and emission wavelengths of 390 and 470 nm, respectively. The surface hydrophobicity was calculated as the slope of a plot of the FI vs. protein concentration.

### Determination of Polypeptide Composition and Profile

The polypeptide composition was determined using the reducing (R) and non-reducing (NR) sodium dodecyl sulfate-polyacrylamide gel electrophoresis (SDS-PAGE) as previously described by Aderinola et al. (33). Briefly, a 2.5% (w/v) sample was prepared with the NR buffer (Tris-HCl buffer, pH 8.0 containing 10%, w/v SDS) or R buffer (Tris-HCl buffer, pH 8.0 containing 10%, w/v SDS and 10%, v/v β-mercaptoethanol). The mixtures were heated at 95°C for 10 min, cooled and then centrifuged at 10,000 × *g* for 10 min. The supernatant (1 μL) was loaded onto 8–25% gradient gels and electrophoresis performed with the Phastsystem™ Separation and Development units according to the manufacturer's instructions (Cytiva, Montreal, PQ, Canada). The gels were stained with Coomassie brilliant blue.

### Determination of Sulfhydryl Group (SH) and Disulfide Linkages (SS)

The SS and free SH contents were determined as described by Tang et al. (34). Protein dispersions (7.5 mg/mL each) were prepared using Tris-Gly buffer (0.086 M Tris, 0.09 M glycine, 0.004 M EDTA, pH 8.0) containing 8 M urea and stirred overnight at room temperature. To obtain the SH content, 1 mL of the stirred protein dispersion was added to 4 mL Tris-Gly buffer and 0.05 mL Ellman's reagent (5,5-dithio-bis-2-nitrobenzoic acid in Tris-Gly buffer), incubated for 20 min at 25^o^C and absorbance measured at 412 nm. To determine total SH content (free SH and reduced SS), 1 mL of the stirred protein dispersion was mixed with 4 mL Tris-Gly buffer and 0.05 mL 2- mercaptoethanol (ME) and incubated for 1 h at 25^o^C. A 10 mL aliquot of 12% (w/v) trichloroacetic acid (TCA) was added to the solution and incubated for another 1 h. The mixture was centrifuged at 5,000 × *g* for 10 min and precipitate resuspended in 5 mL of the TCA solution and centrifuged again (this was performed three times to remove the ME). The final precipitate was suspended in 10 mL of Tris-Gly buffer and 0.04 mL Ellman's reagent was added to 4 mL of the suspension, incubated for 20 min and absorbance taken at 412 nm.

Calculations were made as follows:


(1) $$\begin{array}{r} \mu molSH/g=\left[73.53\times \left(A_{412}\times D\right)/C\right] \end{array}$$

Where A_412_ is the absorbance at 412 nm, C is the sample concentration (mg/mL), D is the dilution factor (5 and 10 for free SH and total SH content, respectively), and the constant 73.53 was calculated from 10^6^/(1.36 × 10^4^), the 10^6^ being conversion from molar to μM/mL and from mg solids to g solids while 1.36 × 10^4^ is the molar absorptivity. The SS content was calculated by subtracting the free SH content from the total SH content and dividing the result by 2.

### Determination of the Surface Charge (Zeta Potential) for the Native Protein and Fractions

Electrophoretic mobility (μ_e_) of homogenous solutions (0.05%, w/v) of the protein samples was measured at room temperature using PALS Zeta Potential Analyzer Ver 5.67 (Brookhaven Instruments Corp., Holtsville, NY) and the zeta potential (ζ, mV) was determined as a function of pH and protein type. The solutions were dispensed in a 1.5 mL cuvette and measurements taken at pH 3.0, 5.0, 7.0 and 9.0 after adjustments based on the prior determined particle size (50–200 nm).


(2) $$\begin{array}{r} \mu_{\text{e}}=\frac{v}{E} \end{array}$$

(3) $$\begin{array}{r} \zeta =\frac{\left(4\pi \eta \right)}{\varepsilon }f\left(\mathrm{Ka}\right).\mu_{\text{e}} \end{array}$$

Where μ_e_ is the migration of particles through an electric field, *v* is the velocity of the particles in the electrical field and *E* is the Electrical field. Zeta potential was calculated from Helmholtz–Smoluchowski equation where ζ is the Zeta Potential, η is the Viscosity of the medium, ε is the Dielectric constant, and f(Ka) is the Debye function. The Smoluchowki approximation *f* (kα) for this study was taken as 1.5.

### Thermal Properties

The effect of heat treatment on the denaturation temperature and enthalpy was determined using a differential scanning calorimeter (DSC Q200, TA Instruments, New Castle, DE). Dry protein flours (95–99 mg) were weighed in pans (TA Instruments, New Castle, DE) and hermetically sealed. The thermal curve was obtained by heating the sample from 40 to 250°C at 10 C/min in a standard DSC cell. Enthalpy of denaturation (Δ*H*) and denaturation temperature (*T*_*d*_) were obtained from the endothermic peaks in the thermograms using Universal Analysis 2000 software (Version 4.5A). The DSC had been calibrated against both sapphire and indium standards and an empty pan was used as reference.

### Scanning Electron Microscope Images

The electron micrographs of dried protein powders were determined in a high-resolution Quanta™ FEG 650 Schottky field emission scanning electron microscope (Hillsboro, OR, USA). Samples were sprinkled over a double-sided carbon tape and fixed on SEM stubs.

### Emulsifying Properties

The oil-in-water emulsions were prepared by mixing protein samples (10, 15, and 20 mg/mL, based on protein content) that were prepared in 0.1 M phosphate buffer at pH 3.0, 5.0, 7.0, or 9.0 with 1 mL of pure canola oil added. The mixture was homogenized at 20,000 rpm for 1 min using the 20 mm non-foaming shaft on a Polytron® PT 3100 homogenizer. Emulsifying activity (EAI) and stability (ESI) index were determined as described by Pearce and Kinsella (35). A 50 μL aliquot of the emulsion was taken from the bottom of the tube at 0 and 10 min after homogenization and then added to 10 mL of 0.1% (w/v) SDS solution. Absorbance of the diluted emulsion was read at 500 nm and calculations done as follows:


(4) $$\begin{array}{r} EAI\;\left(m^{2}/g\right)=2\times \left(2.303xA_{0}xN\right)/\left(cx\phi x10000\right) \end{array}$$

(5) $$\begin{array}{r} ESI\;\left(min\right)=\left(A_{0}/\Delta A\right)/t \end{array}$$

Where, A_0_ is the absorbance of the fresh emulsion dilution at time zero, N is the dilution factor (200), c is the protein concentration per volume (g/ mL), ϕ is the oil volume fraction, ΔA is the change in absorbance 10 min (A_0_) after homogenization (A_0_-A_10_) and t is time interval (10 min).

### Foaming Properties

The foams were prepared by a method described by Aderinola et al. (33). The protein mixtures (10, 15, and 20 mg/mL) were prepared in 0.1 M phosphate buffers at pH 3.0, 5.0, 7., or 9.0 and homogenized at 20,000 rpm for 1 min using a 20 mm foaming shaft on the Polytron® PT 3100 homogenizer (Kinematica AG, Lucerne, Switzerland). The foam capacity and stability were determined as follows:


(6) $$\begin{array}{l} \text{Foam capacity }(\%)\text{ = }(\text{foam volume after whipping/}\\ \text{ initial volume before whipping})\;\times \text{ 100} \end{array}$$

(7) $$\begin{array}{l} \text{Foam stability }(\%)\text{ = }(\text{foam volume after 30 min/}\\ \text{ foam volume after whipping})\;\times \text{ 100} \end{array}$$

### Water Holding and Oil Holding Capacity

WHC and OHC were determined as previously described by Ajibola et al. (30) with slight modifications. A 20 mg/mL sample was prepared by dispersing 0.2 g sample in 10 mL buffers at pH 3.0, 4.0, 5.0, 6.0, 7.0, 8.0, or 9.0 (or oil) contained in a 50 mL pre-weighed centrifuge tube. The dispersion was vortexed for 1 min, allowed to stand for 30 min and then centrifuged at 7,000 × *g* for 25 min at 25°C. The supernatant was decanted, and excess water or oil was drained for 15 min; the gram of water or oil retained per gram of sample was calculated.

### Least Gelation Concentration

LGC was determined as previously described (33). The protein samples were suspended in water or 0.5% (w/v) NaCl at different concentrations (5–25%, w/v) and the mixtures vortexed, placed in a water bath at 95°C for 1 h, cooled under running tap water and stored in the refrigerator (4°C) for 14 h. The LGC is the sample concentration at which the gel did not slip when the tube was inverted.

### Statistical Analysis

Protein aggregates were produced from three separate experiments and then combined for analyses. Samples were then analyzed in triplicates and the mean values subjected to analysis of variance. Significant differences (*p* < 0.05) between mean values were determined by the Duncan's multiple range test using IBM SPSS Statistics for Windows, Version 26.0.

## Results and Discussion

### Protein Content and Yield

Table 1 reveals that protein content was dependent on the fraction size as the lower MW fractions (< 30 and 30–50 kDa) had ≤ 20% while the bigger >50 kDa fraction contained >60% protein. The results suggest that most of the protein aggregates were in the >50 kDa fraction, which indicates successful heat-induced polymerization of the pea proteins. The rationale for analyzing higher molecular weight (HMW) fractions for physicochemical and techno-functional properties is because higher protein content enhances most of the functional properties (36, 37). The lower protein content of the < 50 kDa fractions is indicative of the abundance of low molecular weight (LMW) non-proteins substances. Membrane ultrafiltration produces protein ingredients through application of selective barrier or sieve-like materials to fractionate, purify, and concentrate (38). Since the fractionation protocol was consecutive first with 30 kDa and then 50 kDa, removal of non-protein materials (permeates) at these stages also contributed to the higher protein content of the final retentate (>50 kDa fraction). The LMW (< 50 kDa) proteins probably contained albumin polypeptides, sugars, and secondary metabolites like phenolic compounds (39, 40). The HMW fractions were renamed as FT3, FT5, FT7, and FT9 (fractionated at pH 3.0, 5.0, 7.0, and 9.0, respectively), and used in the comparative study with unfractionated PPC which is a conventional ingredient in the food processing industry. FT5 contained 64.5% protein, which was slightly lower than that of PPC, FT3, FT7, and FT9. The lower protein content at pH 5.0 may be because this is the least point of solubility, hence protein unfolding necessary to enhance aggregation was less. The results are consistent with the higher amounts (gross yield) of < 50 kDa fractions at pH 5 when compared to the other pH values where solubility is higher and hence the propensity to form bigger aggregates is greater. However, yield of the >50 kDa fraction was highest at pH 3.0 and 7.0, which suggest optimal conditions for protein-protein interactions. In contrast, formation of protein aggregates was less efficient at pH 9.0, which is the farthest from the isoelectric point and could be attributed to excessive levels of negative charges. The high density of negative charges at pH 9.0 would have reduced protein-protein interactions during heat treatment, and hence lower yield of the >50 kDa aggregates when compared to pH 3.0, 5.0, and 7.0.

**Table 1:** Protein content and yield of >50 kDa pea protein aggregates isolated by membrane ultrafiltration after heat treatment (100°C) at different pH values.

Samples^*^	**Treatment pH**	**Molecular weight (kDa)**	**^†^Protein content (%)**	**^†^Protein yield (%)**
PPC (control)			68.6 ± 0.05^b^	
PPT1	3.0	< 30	2.24 ± 0.96^h^	1.14 ± 0.05^e^
PPT2		30–50	6.24 ± 0.36^g^	1.30 ± 0.05^e^
PPT3 (FT3)		>50	68.4 ± 0.02^b^	35.2 ± 4.53^a^
PPT4	5.0	< 30	14.1 ± 0.41^e^	1.85 ± 0.05^e^
PPT5		30–50	16.3 ± 0.24^e^	2.80 ± 0.04^e^
PPT6 (FT5)		>50	64.5 ± 0.04^c^	22.7 ± 1.06^c^
PPT7	7.0	< 30	11.4 ± 1.09^f^	0.55 ± 0.04^f^
PPT8		30–50	10.8 ± 1.10^f^	0.25 ± 0.02^f^
PPT9 (FT7)		>50	73.8 ± 1.10^a^	27.9 ± 3.53^b^
PPT10	9.0	< 30	10.4 ± 0.81^f^	0.66 ± 0.01^f^
PPT11		30–50	20.4 ± 0.50^d^	1.39 ± 0.33^e^
PPT12 (FT9)		>50	71.2 ± 0.17^a^	15.0 ± 0.44^d^

a−h *For each column, different letters indicate significant differences (p ≤ 05)*.

* *Untreated pea protein concentrate (PPC) and ultrafiltration fractions (FT1–FT12)*.

† *Mean of triplicate determinations ± standard deviation*.

### Sulfhydryl Groups (SH) and Disulfide Linkages (SS)

During heat treatment, proteins undergo structural and conformational changes namely unfolding (denaturation), exposure of hidden reactive groups and SCAA, protein-protein interactions *via* hydrophobic interaction and the formation of new disulfide linkages through reaction between two SH side chains of two cysteine residues. Results as shown in Table 2 reveal that the FT3 and FT9 had similar total SH contents as the PPC while FT5 was significantly lower and FT7 had the highest value. The results had direct relationship with the protein contents of the samples. Similarly, the exposed SH result showed lowest value for FT5, which suggest that at pH 5.0 most of this group were situated away from the protein aggregate surface when compared to the aggregates formed at pH 3.0, 7.0, and 9.0. The FT5 also contained significantly (*p* < 0.05) lower number of SS, which is consistent with the smaller number of total SH groups when compared to the other protein aggregates. The presence of exposed SH has been shown to positively influence protein solubility (41, 42), which is an important functionality in promoting the use of proteins as food ingredients. Exposed SH in a protein could be an index for conformational changes such as structural unfolding or the cleaving of the SS bonds during processing. Therefore, the results suggest that the FT5 is a less unfolded protein aggregate than the FT3, FT7, and FT9. Previous works have also suggested that a shift toward alkalinity increases the total number of SS groups, due to formation of more intra and intermolecular bonds during SH/SS interchange reactions, which leads to reductions in number exposed SH (43, 44). This may explain the significant decrease in exposed SH observed in this work for FT9, which was formed at pH 9.0 when compared to pH 7.

**Table 2:** Sulfhydryl groups and surface hydrophobicity (H_o_) of pea protein aggregates isolated by membrane ultrafiltration after heat treatment (100°C) at different pH values.

	**^†^Sulfhydryl groups (μmol/g)**			**^†^Ho**
Samples^*^	**Total sulfhydryl**	**Exposed sulfhydryl**	**Disulfide bonds**	
PPC	68.81 ± 0.30^b^	18.28 ± 0.00^b^	25.26 ± 0.13^ab^	11.22 ± 0.24^c^
FT3	69.27 ± 0.30^b^	20.16 ± 0.22^ab^	24.55 ± 0.17^b^	4.97 ± 0.32^e^
FT5	44.50 ± 0.20^c^	8.40 ± 0.15^c^	18.05 ± 0.04^c^	8.73 ± 0.15^cd^
FT7	75.23 ± 0.06^a^	22.61 ± 0.08^a^	26.51 ± 0.04^a^	58.9 ± 0.44^b^
FT9	69.33 ± 0.25^b^	19.36 ± 0.01^b^	25.00 ± 0.12^ab^	278.7 ± 0.10^a^

a−d *For each column, different letters indicate significant differences (p ≤ 05)*.

* *Untreated pea protein concentrate (PPC) and the >50 kDa protein aggregates formed at pH 3.0 (FT3), 5.0 (FT5), 7.0 (FT7), and 9.0 (FT9)*.

† *Mean of triplicate determinations ± standard deviation*.

### Surface Charge and Hydrophobicity (Ho)

**Figure 1:**
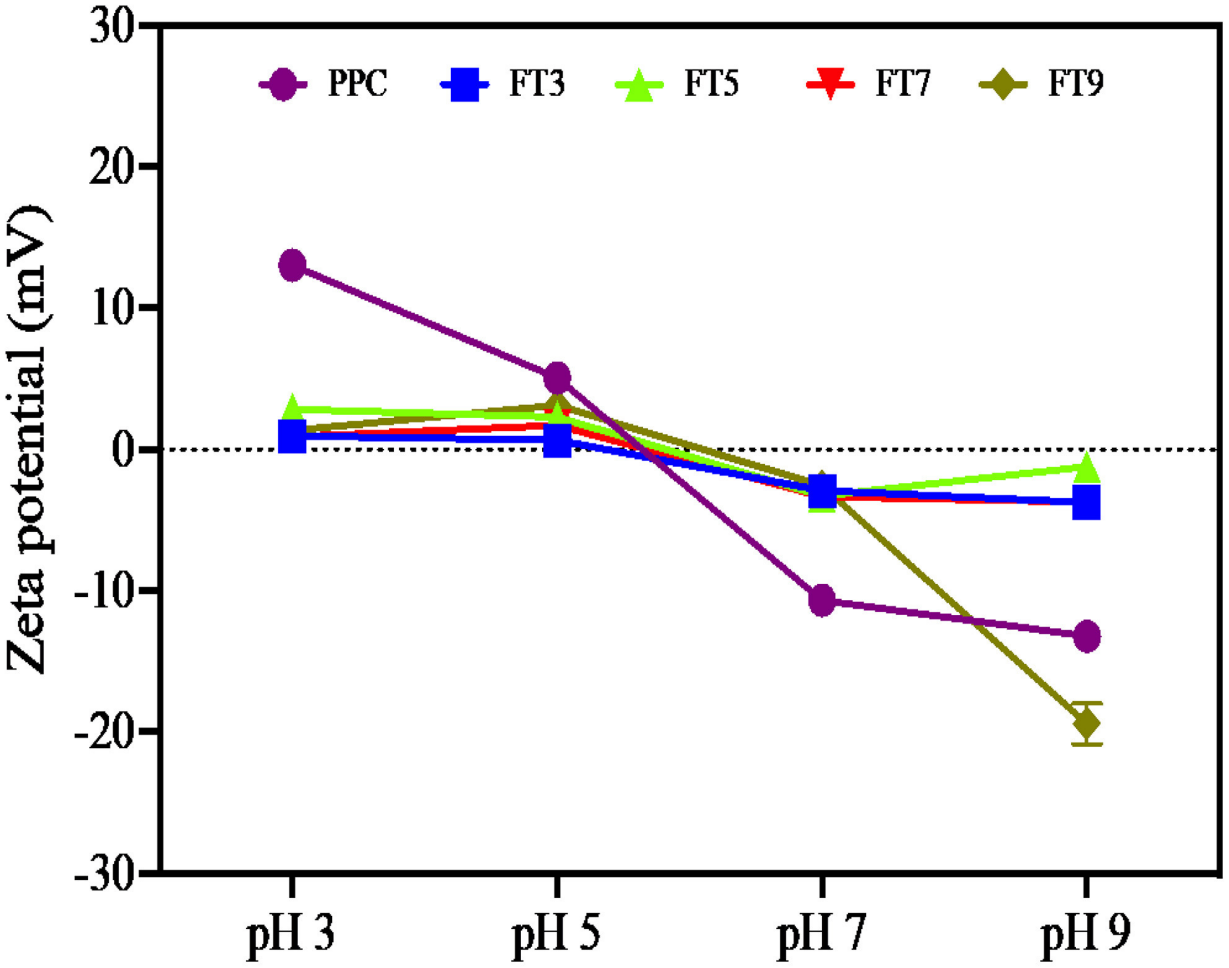
Surface charge of the native protein (PPC) and isolated fractions at pH 3.0, 5.0, 7.0, and 9.0. measured as the zeta potential (mV). PPC was heated at 100°C and varying pH, centrifuged, and the supernatant passed consecutively through 30 and 50 kDa membranes. The final retentate from the 50 kDa membrane was collected as FT3, FT5, FT7, and FT9 for heat treatments at pH 3.0, 5.0, 7.0, and 9.0, respectively.

Surface charge and hydrophobicity of proteins influence the solubility and subsequently, other functional properties such as foaming, thickening, gelation, and emulsification properties. The surface charge on proteins is the intrinsic effect of ionizable groups (amino and carboxyl) present and the average charge is measured as the zeta potential (17). The result as shown in Figure 1 reveals that the net surface charge of PPC and the fractions are relatively low under which conditions the electrostatic repulsive forces are low and incidence of protein to protein interactions is eminent (14). Notwithstanding, surface charge of PPC was highest at pH 3.0 and 5.0 (13 and 14 mV, respectively), and at pH 9.0, which suggests the presence of electropositive and negative charges on the surface of the native protein at acidic and alkaline pH, respectively. The surface charge of other pea protein isolates extracted by different methods was reported to be ~21 mV at pH 7.0, which is low when compared to other legumes (17). Similarly, Hayati Zeidanloo et al. (45) showed that variations exist in surface charge of pea protein prepared by different methods (between −21.73 and 24.96 mV at pH 7.0). On the contrary, another study reported that the surface charge of lab prepared field protein isolate was −44.2 mV and higher than that of kidney bean (40.0 mV) and amaranth protein isolates (37.3 mV) (46). Cui et al. (43) also reported high surface charge of four yellow pea protein cultivar at pH 3.0–8.0 ranging between −30 and 30 mV. The study suggested a direct relationship between the surface charge of the proteins and solubility, which was not the case with the present study. Apart from FT9, which had relatively high surface charge at pH 9.0 (−19.4 mV), the aggregates had low surface charge at both low and high pH (~-2 to −4 mV). Although not statistically significant (*p* > 0.05), FT3 and FT7 had a slightly higher charge than FT5 at high pH values. Bogahawaththa et al. (15) showed that heat treatment of pea protein at 121°C (pH 6.8–7.5) improved the surface charge by at least 5% but a decrease of at least 14% occurred after ultra-heat treatment (140°C). The referenced study also showed that the surface charge of supernatants derived from most of the heated protein solutions was lower than the those measured from the bulk samples by at least 8%. In this study, supernatants from protein solutions were analyzed for surface charge and may explain the reason for the observed low values because the isolated proteins consisted mainly of aggregates. Factors that could impact the surface charge of proteins includes amino acid composition, protein conformation, environment (ionic strength, pH, and temperature of solvent) and protein concentration (17, 39). Table 2 also shows that FT7 and FT9 had significantly (*p* < 0.05) higher H_o_ values than PPC, FT3, and FT5, which suggest a more open protein conformation at pH above the isoelectric point. The results indicate that at pH 7.0 and 9.0, the increased net charge on proteins may have reduced protein-protein interactions accompanied by dissociation of the aggregates to expose hydrophobic patches (47). The FT3 and FT5 had similar H_o_ as the PPC, which indicates that at pH 3.0 and 5.0, the protein aggregates had conformations with most of the hydrophobic groups shifted into the core and away from the hydrophilic environment (48). Hydrophobicity means “water fearing,” which implies that the higher the surface hydrophobicity of a protein, the lower the solubility and vice versa. However, contrary views suggesting a positive relationship between surface hydrophobicity and solubility have been reported (49).

### Polypeptide Composition and Profile

**Figure 2:**
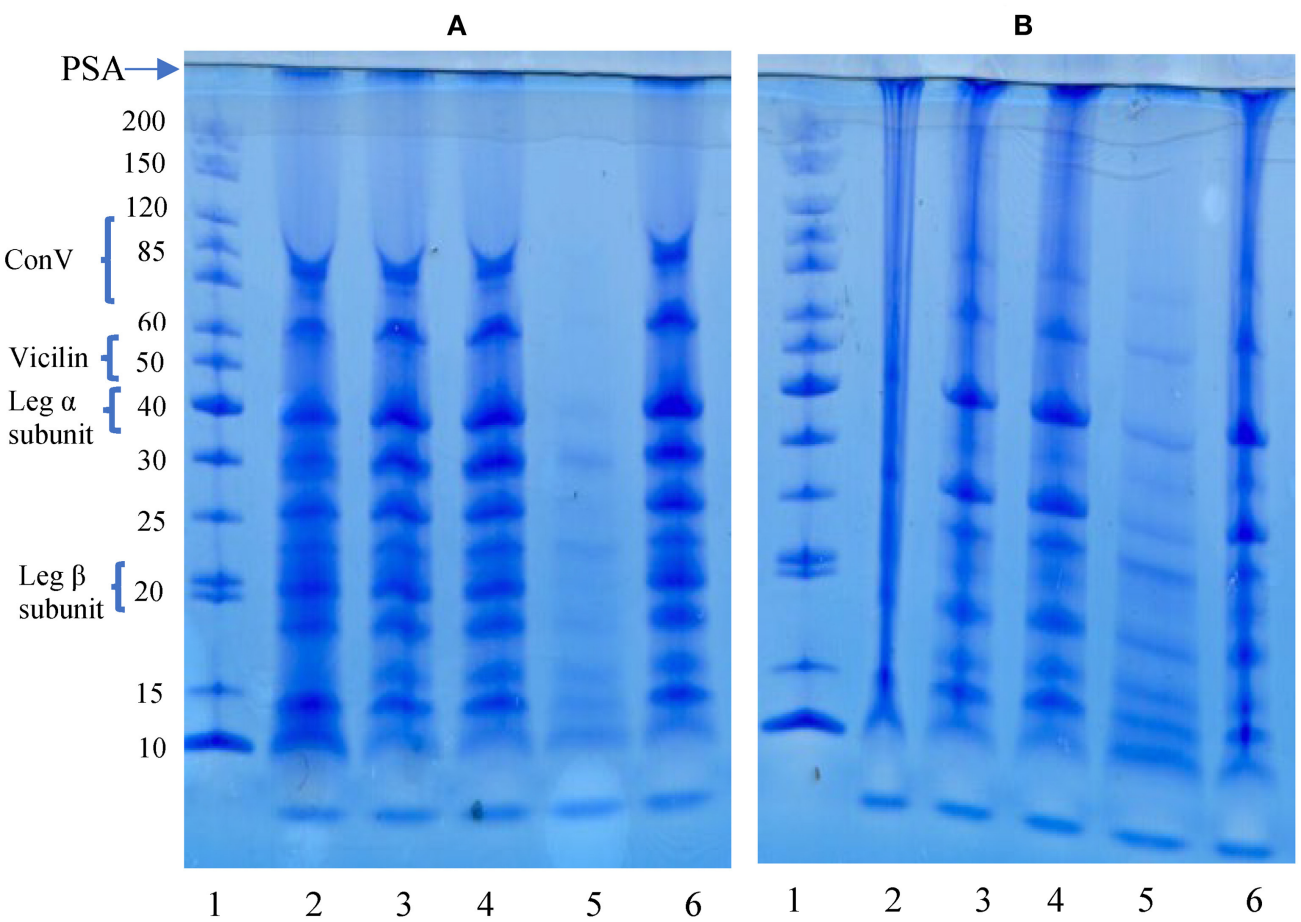
SDS-PAGE under reducing **(A)** and non-reducing **(B)** conditions. PSA, point of sample application. Lanes: 1, standard proteins; 2, pea protein concentrate (PPC); 3, FT3; 4, FT5; 5, FT7; 6, FT9. PPC was heated at 100°C and varying pH, centrifuged and the supernatant passed consecutively through 30 and 50 kDa membranes. The final retentate from the 50 kDa membrane was collected as FT3, FT5, FT7, and FT9 for heat treatments at pH 3.0, 5.0, 7.0, and 9.0, respectively.

Polypeptide composition of the aggregates and native pea protein was determined using reducing and non-reducing gel electrophoresis, which separation is based on molecular weight. Figure 2 shows that polypeptide sizes ranged from ~8.0–200 kDa for both gels while bigger protein aggregates (≤200 kDa) were immobilized at the point of sample application (PSA) and did not enter the gel, especially under non-reducing condition. The reduction in intensity of the >200 kDa band (PSA) under reducing condition is an indication that some of the protein aggregates that did not enter the gel under non-reducing electrophoresis were held together by disulfide bonds. The profile revealed a wide variety of polypeptides attributed to be constituents of vicilin (7S) and legumins (11S). Under reducing condition, the PPC produced 11 bands, which indicates dissociation of 11S fraction in the presence of a reducing agent as previously reported (50–52). The polypeptide profiles of the isolated aggregates were like that of PPC, which confirms heat-induced interactions of the native proteins led to polymer formation.

### Scanning Electron Microscope Images

**Figure 3:**
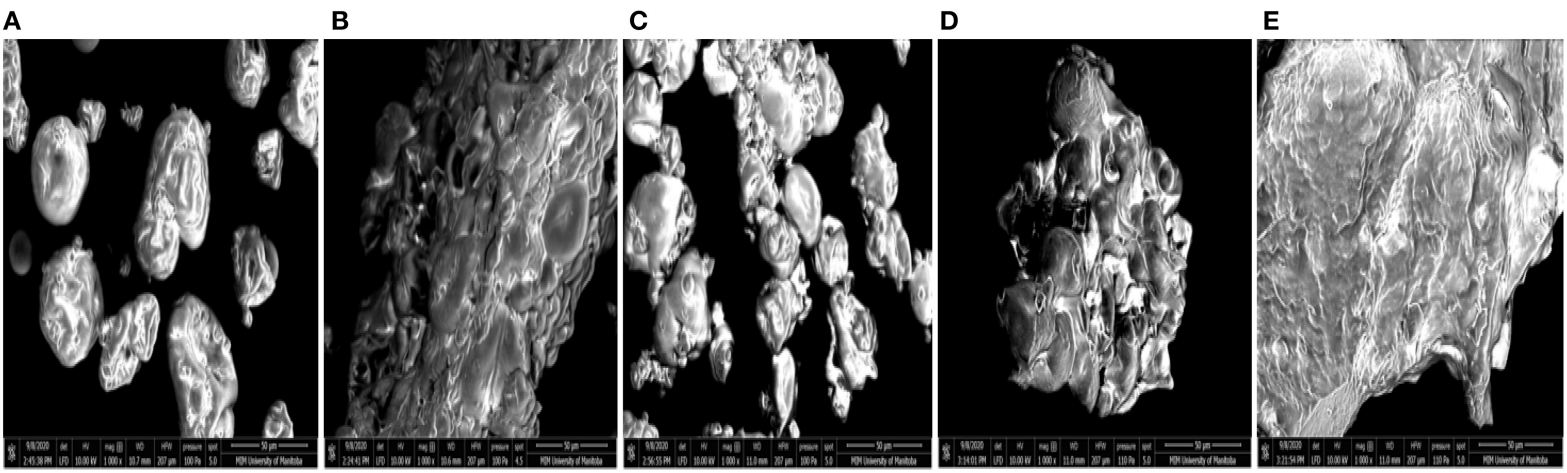
Scanning electromicrographs of: **(A)** pea protein concentrate (PPC), **(B)** FT3, **(C)** FT5, **(D)** FT7, and **(E)** FT9 at 1,000× magnification. PPC was heated at 100°C and varying pH, centrifuged, and the supernatant passed consecutively through 30 and 50 kDa membranes. The final retentate from the 50 kDa membrane was collected as FT3, FT5, FT7, and FT9 for heat treatments at pH 3.0, 5.0, 7.0, and 9.0, respectively.

SEM provides information about the surface topography, morphology and composition by the detecting electrons scattered on the surface of the protein (53). PPC contains large amounts of 11S and 7S proteins, which are represented in different sizes (54). The microstructure of the native pea protein and that of the isolated protein aggregates are shown in Figures 3A–E. The images revealed that heat treatment at different pH values produced proteins with varying morphologies. PPC showed a mixture of distinct spherical and wrinkled shapes, which is characteristic of high levels of folded native proteins. FT3, FT7, and FT9 were amorphous floating mass that showed greater aggregation and network formation completely different from the native protein form. FT3 (Figure 3B) and FT9 (Figure 3E) showed more signs of network formation than FT5 (Figure 3C) and FT7 (Figure 3D). FT5 showed distinct aggregate forms with reduced level of continuous network formation but was still morphologically different from PPC.

### Thermal Properties

Thermal properties reflect protein transition from one state to another (i.e., native to denatured state) under heat application and is an index for temperature-induced protein unfolding and thermal stability (51). As shown in Table 3, the T_o_ and T_d_ reported for PPC correspond with values reported by Oliete et al. (52) for native pea globulins at 65.11 and 76.66°C respectively. However, some previous works have reported T_o_ and T_d_ values of ~70 and 80°C, respectively for laboratory prepared native pea proteins (55, 56). Reducing heating rate from 10 to 5°C/ min was reported to decrease T_o_ and T_d_ by 4°C but ΔH_d_ was not affected (51). The T_o_ explains structural unfolding of the polypeptides and protein, T_d_ indicates the heat stability of the proteins and ΔH_d_ represents the enthalpy changes that occur during the denaturation process and reflects the extent of ordered structure of a protein (51). This means PPC had less ordered protein structure prior to the thermal treatment, which could also suggest the presence of partially denatured protein in the untreated PPC. In contrast, the isolated protein aggregates all had significantly (*p* < 0.05) higher thermal properties that increased as the environment during thermal treatment was changed from pH 3.0 to pH 9.0. This means the heat and pH treatments produced polypeptides with more compact tertiary structure and stronger protein-protein interactions than the PPC (44). The exothermic reaction also means weakening and disruption of hydrophobic bonds (57). Therefore, the higher ΔH_d_ values obtained for the aggregated proteins also confirm the presence of a more extensive network of hydrophobic interactions when compared to the untreated PPC. The low ΔH_d_ values indicate that the protein aggregates were held together mainly by the non-covalent hydrophobic bonds (57). The increases in T_o_ and T_d_ values from pH 3.0 (FT3) to pH 9.0 (FT9) are consistent with changes in surface hydrophobicity (Table 2), which further supports the role of hydrophobic interactions as the main interactive forces responsible for formation of the protein aggregates.

**Table 3:** Thermal properties of pea protein aggregates isolated by membrane ultrafiltration after heat treatment (100°C) at different pH values.

Samples^*^	**Onset temperature (T_**o**_)**°**C^†^**	**Maximum temperature (T_**d**_)**°**C^†^**	**ΔH (J/g of sample)^†^**
PPC	66.50 ± 0.20^d^	74.45 ± 1.50^e^	0.06 ± 0.01^c^
FT3	89.15 ± 0.04^c^	124.30 ± 0.5^d^	1.43 ± 0.04^b^
FT5	160.12 ± 6.20^b^	190.66 ± 1.56^b^	2.47 ± 0.33^a^
FT7	176.56 ± 3.30^a^	206.33 ± 0.17^a^	1.87 ± 0.15^a^
FT9	171.36 ± 0.5^a^	203.17 ± 0.38^a^	1.58 ± 0.61^a^

a−e *For each column, different letters indicate significant differences (p ≤ 05)*.

* *Untreated pea protein concentrate (PPC) and the >50 kDa protein aggregates formed at pH 3.0 (FT3), 5.0 (FT5), 7.0 (FT7), and 9.0 (FT9)*.

† *Mean of triplicate determinations ± standard deviation*.

### Protein Solubility

**Figure 4:**
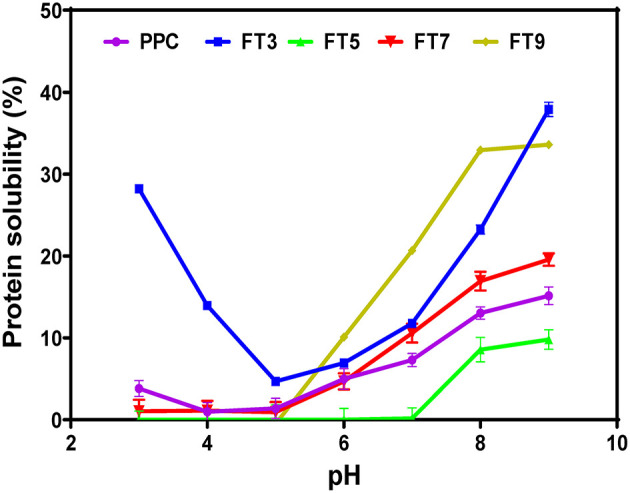
pH solubility profile of pea protein concentrate (PPC) and >50 kDa pea protein aggregates. PPC was heated at 100°C and varying pH, centrifuged and the supernatant passed consecutively through 30 and 50 kDa membranes. The final retentate from the 50 kDa membrane was collected as FT3, FT5, FT7, and FT9 for heat treatments at pH 3.0, 5.0, 7.0, and 9.0, respectively.

Protein solubility is a measure of equilibrium between protein-protein and protein-solvent interactions and is a pivotal property with respect to protein utilization in food processing, which depends largely on hydration properties of the ingredients. These properties are influenced by the composition, sequence, conformation, and surface charge of the protein moiety. Protein functionality especially emulsification, gelation and foaming largely depends on solubility (58). The results in Figure 4 revealed that FT3 had a superior solubility of ~60% in comparison with PPC (~30%) at both acidic and alkaline environments. PPC and the other fractions exhibited improved solubility toward alkaline pH, which agrees with reports on native and heat-treated legumes (58). Apart from FT5 which had the lowest solubility, all the other fractions had better solubility than PPC. The reason for the low solubility of the FT5 could be attributed to isolation close to the isoelectric point of the protein, which would have produced compact and less flexible protein aggregates as evident in Figure 3C. In contrast, the FT3, FT7, and FT9 had superior solubility due to the presence of repulsive electrostatic interactions within the environment during heat-induced aggregation, hence more flexible and less compact protein structures (Figures 3B,D,E). Increased solubility occurs with the presence of net positive and negative charges, which introduces electrostatic repulsive forces between the protein molecules and attraction between the protein and water molecules. However, only FT3 displayed the U-shape solubility profile that is typical of most plant proteins. Although heating and aggregation impair solubility and other protein functionalities, heat treatment for longer periods has been shown to produce aggregates with improved solubility (15).

### Emulsifying Activity and Stability Index

**Figure 5:**
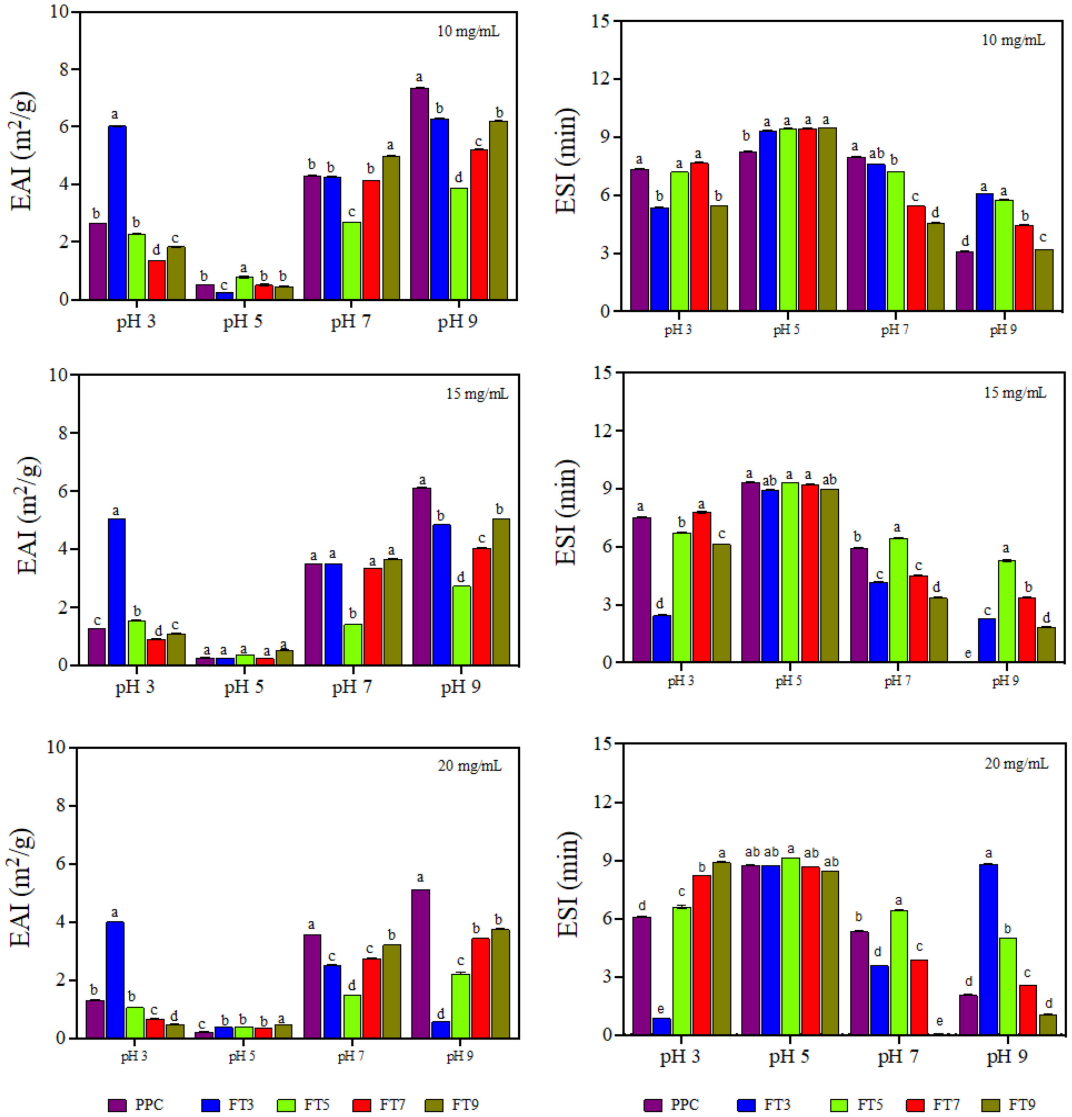
Emulsifying activity index (EAI, m^2^/g) and emulsion stability index (ESI, min) of pea protein concentrate (PPC) and >50 protein aggregates. PPC was heated at 100°C and varying pH, centrifuged and the supernatant passed consecutively through 30 and 50 kDa membranes. The final retentate from the 50 kDa membrane was collected as FT3, FT5, FT7, and FT9 for heat treatments at pH 3.0, 5.0, 7.0, and 9.0, respectively. For each plot, bars with different letters have different mean values (*p* < 0.05).

Emulsifying activity index (EAI, m^2^/g) is the area of oil/water interface that can be emulsified per unit weight of protein and is dependent on the adsorption of proteins onto the interfacial layer (17). The EAI profile (Figure 5) looked very similar to the protein solubility profile showing pH dependency with the lowest activity observed at pH 5.0 (58). The higher EAI at pH 9.0 is consistent with a previous report by Chang et al. (48). The results confirm the important role of increased protein-water interactions (solubility) in enhancing emulsification of oil droplets (19). Similarly, EAI was dependent on the sample type and protein concentration (*p* < 0.05). FT3 had slightly better EAI than all the fractions and the control by ~6%, which indicates greater unfolding and dissociation during emulsification that led to increased surface activity and enhanced adsorption at the oil-water interface. The low EAI of FT5 could be attributed to a combination of the compact structure as revealed by SEM (Figure 3C) and low solubility, which reduced ability to form interfacial membranes around the oil droplets.

The current study revealed that the relationship between emulsifying activity of a protein and the physicochemical properties is complex and dependent on many other factors as previously suggested (48). EAI declined with increasing protein concentration (10 > 15 > 20 mg/mL), which indicates molecular crowding and increased viscosity of the continuous phase to prevent adequate formation of the interfacial membrane. Emulsifier concentration influences emulsification activity because of the amount of protein that covers the interfacial layer and protein concentration could reach a saturation point at higher levels (59). Chen et al. (60) reported increase in the emulsification properties of thermally treated pea protein when the concentration in the emulsion increased to 10 mg/mL but above this concentration, the interfacial saturation point of the protein was reached. However, Aziz et al. (61) reported that increasing corn protein concentration (0.1–2%, w/v) improved the EAI, which suggests that the type of protein is also important.

Emulsion stability index (ESI) is the measure of the ability of the formed emulsion to resist changes to the structure over time (62). ESI is an indicator of the shelf life and stability of the food product against external stressors of the environment, freeze thaw and transportation but with dependence on the characteristics of the interfacial layer (17). The ESI result revealed pH dependency but was not influenced by protein concentration nor sample type (Figure 5). The emulsions prepared at pH 5.0 were more stable by ~30% and the least stability was obtained at pH 9.0, which reflects the role of protein charge. This is because the proteins have least charge at pH 5.0, which would have reduced protein-protein repulsion and lead to formation of stronger interfacial membranes. In contrast, the high charge density at pH 9.0 led to strong protein-protein repulsions, hence weak interfacial membranes were formed.

### Foam Properties

**Figure 6:**
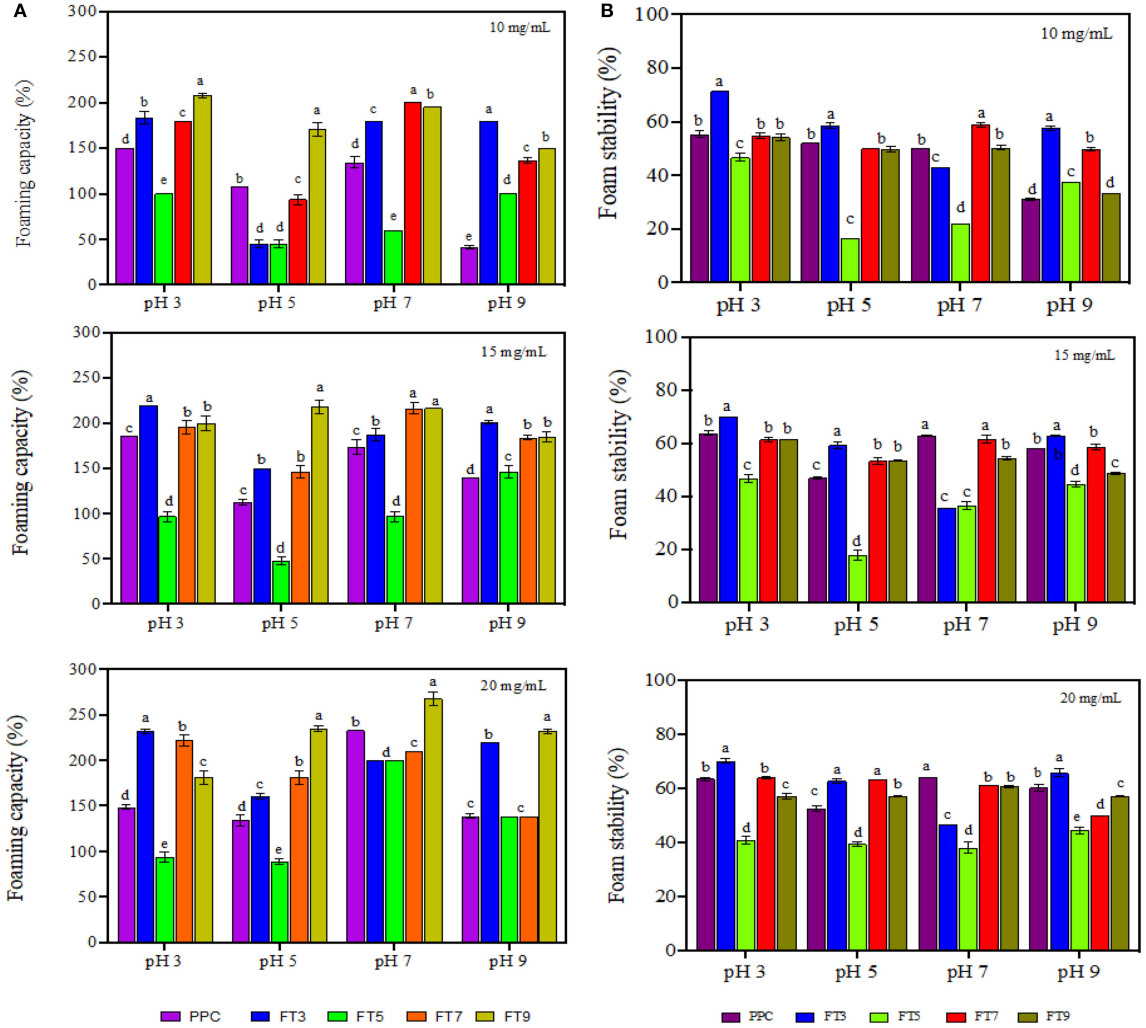
**(A)** Foaming capacity and **(B)** foam stability of pea protein concentrate (PPC) and >50 protein aggregates. PPC was heated at 100°C and varying pH, centrifuged and the supernatant passed consecutively through 30 and 50 kDa membranes. The final retentate from the 50 kDa membrane was collected as FT3, FT5, FT7, and FT9 for heat treatments at pH 3.0, 5.0, 7.0, and 9.0, respectively. For each plot, bars with different letters have different mean values (*p* < 0.05).

Foams are made of air bubbles dispersed in a continuous phase which could be a liquid (e.g., whipped cream) or a solid (e.g., marshmallows). Food proteins are very popular foaming agents in the food industry because of their ability to adsorb at the air-water interface of the foam followed by rapid reduction of the interfacial tension and formation of cohesive film around the dispersed air bubbles (63). Foam properties are measured using the foam capacity (foamability) and foam stability. Foamability is the foam volume after introduction of a gas and reflects the level of air in the dispersed phase while foam stability is the rate at which the foam volume decreases over time mostly due to effect from external stressors and gravity (64). As shown in Figure 6, FT3 had significantly (*p* < 0.05) higher foaming capacity by ~10% in comparison to PPC while FT5 had a decline in activity by 31%. Foaming capacity of FT7 and FT9 was not significantly different from PPC but in general the values increased at a higher protein concentration (20 mg/mL) and at pH 3.0. Chao and Aluko (65) had earlier reported improved foam capacity at high concentrations of heat pretreated pea protein due to increased availability of polypeptide chains. However, the study also showed that foam capacity reduced when the pea protein was treated at 90–100 °C. Furthermore, findings from Chao and Aluko (65) also suggest pH-dependent foam capacity and treatments at pH 7.0 had better capacity than pH 3.0 and 5.0 resulting from increased flexibility and net charge. Statistically, there were significant differences (*p* < 0.05) in the foam capacity at 10, 15, and 20 mg/mL protein concentrations, with values influenced by sample type, pH, and protein concentrations. This finding is consistent with literature as the ability of protein to foam depends on the nature of the protein such as the surface properties (surface hydrophobicity and charges) and the processing conditions such as pH, temperature, ionic strength, and shear force (66). Foamability of the protein aggregates had a strong relationship with solubility, which corresponds to reports by Shevkani et al. (67) that cowpeas protein with higher solubility was associated with superior foaming capacity.

From Figure 6, foam stability was observed to be dependent on the protein concentration, sample type and pH (*p* < 0.05) with the best stability obtained at pH 3.0 and 20 mg/mL. FT3 and FT7 had ~7% foam stability increases in comparison with PPC but there was no significant difference between FT5, FT9, and PPC. The reason could be that the greater presence of surface charge (electrostatic repulsive forces) at pH 3.0 and 7.0 facilitated increased repulsions between the encapsulated air bubbles, hence better foam stability when compared to pH 5.0 (17). A previous work has also shown that foam stability improved toward alkalinity in native and heat-treated legumes with better stability at pH 7.0 (58).

### Water and Oil Holding Capacity (WHC and OHC)

**Figure 7:**
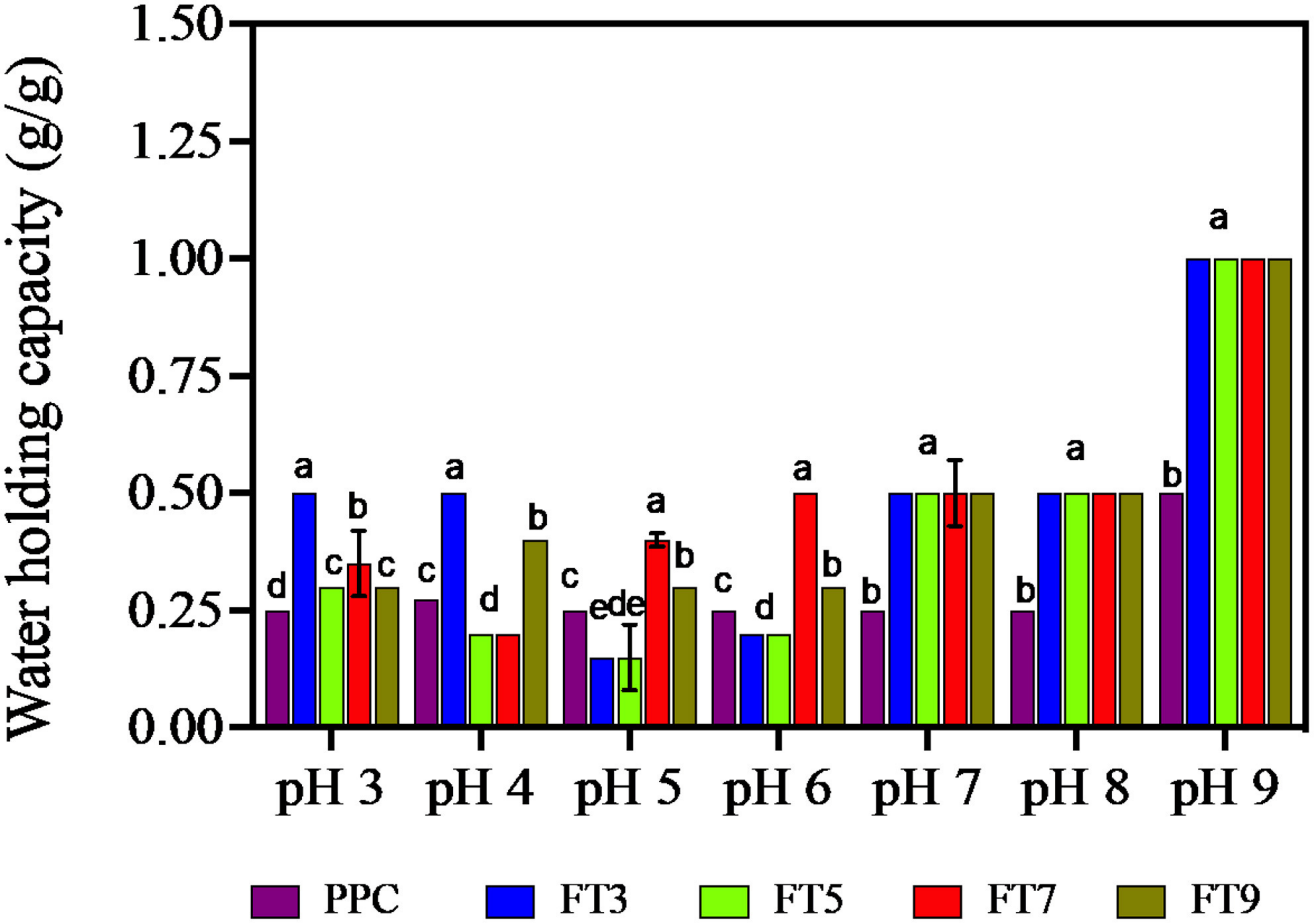
Water holding capacity (g/g) of pea protein concentrate (PPC) and >50 protein aggregates. PPC was heated at 100°C and varying pH, centrifuged and the supernatant passed consecutively through 30 and 50 kDa membranes. The final retentate from the 50 kDa membrane were collected as FT3, FT5, FT7, and FT9 for heat treatments at pH 3.0, 5.0, 7.0, and 9.0, respectively. Bars with different letters have different mean values (*p* < 0.05).

WHC is the amount of water that 1 g of the protein can hold to prevent expulsion from within the matrix (14). OHC shows how much oil is entrapped within the protein matrix and influences flavor retainment and mouth feel of the food product (14). Factors that influence WHC and OHC are the surface properties of the protein (hydrophobic interactions, surface charges, covalent, and non–covalent bonds), protein structure (molecular weight, pores, and capillary sizes) and the surrounding environment (pH, ionic strength, and temperature) (30). As shown in Figure 7, except for FT5, the isolated protein aggregates had ~16% improvement in WHC than the PPC at acidic pH and ~50% at neutral and alkaline pH. The protein aggregates had similar WHC values at pH 7.0, 8.0, and 9.0 but were distinct from the PPC. Similarly, FT3, FT7, and FT9 had significantly higher (*p* < 0.05) OHC (~100%) in comparison with PPC and FT5 (Table 4). The OHC of the samples and control declined in the order FT9 > FT3 > FT7 > FT5 > PPC. WHC indicates that the protein aggregates may be useful in products where good interactions with water is required, such as in soups and gravies while their OHC values point to usefulness in baked goods.

**Table 4:** Oil holding capacity (g/g) and least gelation concentration (LGC) of pea protein aggregates isolated by membrane ultrafiltration after heat treatment (100°C) at different pH values.

Samples^*^	^*^Oil holding capacity (g/g)	^**^LGC without NaCl (%)	^**^LGC with 0.5% NaCl (%)
PPC	0.23 ± 0.00^c^	18	16
FT3	0.64 ± 0.04^b^	25	18
FT5	0.34 ± 0.00^d^	22	20
FT7	0.62 ± 0.01^b^	22	16
FT9	0.87 ± 0.02^a^	25	18

* *Mean ± standard deviations of triplicate determinations*.

** *Mean of triplicate determinations. Letters a-e represents statistical significance at p ≤ 05. For each column, different letters indicate significant differences (p ≤ 05). Untreated pea protein concentrate (PPC) and the >50 kDa protein aggregates formed at pH 3.0 (FT3), 5.0 (FT5), 7.0 (FT7), and 9.0 (FT9)*.

### Least Gelation Concentration (LGC)

Food gels are three-dimensional structures, which are held together by non-covalent interactions (van Der Waals forces, hydrophobic interactions, hydrogen bond, electrostatic forces) and the covalent sulfhydryl/disulfide linkages. The indices for a superior gel quality are hardness, paste viscosity and minimum gelation concentrations (68). Proteins with superior LGC require less concentration to form high quality gels. Gelation kinetics of soybean protein aggregates was shown to be faster and the gel structures more homogenous although the gel strength was not different when compared to the native protein (69). However, in this study, heat treatment impaired LGC as the PPC performed better with a lower LGC value than the aggregated proteins (Table 4). The results suggest that when compared to the untreated PPC, increased polymer size caused by heat treatment led to poor unfolding ability of the protein aggregates, which is required for protein network formation. However, addition of 0.5% (w/v) NaCl led to improved LGC, but this effect was more pronounced for the isolated protein aggregates than the untreated PPC. For example, while LGC improved by only 2% for the PPC, the presence of NaCl led to up to 7% reduction in amount of aggregated proteins needed to form a gel. As expected, the highly compact structure of the FT5 may have reduced the effect of NaCl addition as only a 2% reduction was achieved when compared to 6–7% for other protein aggregates. The positive effect of NaCl confirms that the protein aggregates are held together mostly by non-covalent interactions, which were readily interrupted to produce protein units with a more flexible structural conformation, thus favoring stronger network formation. The monovalent NaCl ions could have also influenced gelation through screening of repulsive forces on the protein surface, which enhanced protein-protein interactions (69).

## Conclusion

Findings from this study gave valuable information about the structural and functional changes that occurred when pea protein was modified under heat treatment at varying pH conditions. As revealed by SEM, the effect of pH during heat treatment was pronounced because FT3, FT7, and FT9 showed a more flexible protein aggregate arrangement and network formation, and for FT9 at pH 9, higher surface charge, which translated to better functional properties when compared to FT5. The protein aggregates were held together by non-covalent interactions, therefore addition of NaCl was able to increase efficiency of gel formation. Overall, heat treatment at pH 3.0 produced >50 kDa aggregates with better solubility at acidic pH as well as superior emulsifying and foaming properties than similar protein aggregates produced at pH 5.0, 7.0, and 9.0. The outcome of this research may benefit the food industry in the production of novel pea protein ingredients for use in baked goods, beverages, soups, salad dressings and foam products like ice cream, meringue, and marshmallows. A noted drawback and limitation of the study is that the distinct morphology of individual aggregates could not be observed by the SEM. Heat induced aggregation of globular proteins leads to the formation of primary and secondary aggregates with different morphologies and each structure presents a unique functionality in food applications. Therefore, future studies are suggested to characterize the structure-function relationship of the different aggregates. Also, comparative studies with heat-induced aggregates formed from other standard proteins like dairy and soybean are required using the same processing conditions.

## Data Availability Statement

The original contributions presented in the study are included in the article/supplementary material, further inquiries can be directed to the corresponding author/s.

## Author Contributions

RA: conceptualization, funding, supervision, and review and editing. NA: analysis, investigation, and original draft. Both authors contributed to the article and approved the submitted version.

## Funding

The authors acknowledge support from the Natural Sciences and Engineering Council of Canada (NSERC), funding reference number RGPIN 2018-06019.

## Conflict of Interest

The authors declare that the research was conducted in the absence of any commercial or financial relationships that could be construed as a potential conflict of interest.

## Publisher's Note

All claims expressed in this article are solely those of the authors and do not necessarily represent those of their affiliated organizations, or those of the publisher, the editors and the reviewers. Any product that may be evaluated in this article, or claim that may be made by its manufacturer, is not guaranteed or endorsed by the publisher.
